# Supplementary material for: Implementation of rapid and frequent SARS-CoV2 antigen testing and response in congregate homeless shelters
Source: PLoS One. 2022 Mar 10;17(3):e0264929. doi: 10.1371/journal.pone.0264929 (PMC8912252; doi:10.1371/journal.pone.0264929)
Supplement: S1 Appendix — (DOCX) [file pone.0264929.s001.docx]

The population of residents who participated at least once was slightly older, albeit not significantly, than the population that did not participate (median 45.5 and 42 years, n=348 and 483, *p*=0.06 on a Mann-Whitney U test), and their gender distribution was not significantly different (n=213 and 276, p=0.73 on a chi-squared test).

The age of the shelter staff population who tested at least once (n = 232, mean 44.9, 95% CI [42.7 47.1]) was similar to the total staff population age (mean 43.6 years). Slightly more female staff tested at least once (n = 6 shelters, mean 58.3%, 95% CI [46.3 70.2]), compared to the total staff population (n = 6 shelters, mean 51.5%, 95% CI [43.2  59.8]).
