# Supplementary material for: Implementation of rapid and frequent SARS-CoV2 antigen testing and response in congregate homeless shelters
Source: PLoS One. 2022 Mar 10;17(3):e0264929. doi: 10.1371/journal.pone.0264929 (PMC8912252; doi:10.1371/journal.pone.0264929)
Supplement: Attachment — Submitted filename: Review PLOS (2).docx [file pone.0264929.s003.docx]

After the iteration process, you have scaled up testing and advised study participants to do self-testing.

1. There is no mention of any appraisal training through online mode to them.

2. There is no mention of the number of tests done by participants(self swab samples) & their results. Authors should give separate details of the number of these self–swab tests and their results(testing needs proper acumen).

3. What was the time elapsed in the handover of self swab assays to the recorder?

4. Can you give us the rationale behind twice-a-week testing?
