# Supplementary material for: Implementation of rapid and frequent SARS-CoV2 antigen testing and response in congregate homeless shelters
Source: PLoS One. 2022 Mar 10;17(3):e0264929. doi: 10.1371/journal.pone.0264929 (PMC8912252; doi:10.1371/journal.pone.0264929)
Supplement: Attachment — Submitted filename: PONE-D-21-13440-ResponseToReviewers.docx [file pone.0264929.s005.docx]

***Reviewer #1:***

***May describe in more detail about the ethical issues. What do you mean by 'public health surveillance' exemption?***
We have added language to the Ethics Statement in the Methods to clarify public health surveillance exemption.

***In the discussion may consider the varying impact of the effectiveness of rapid and frequent testing depending on the stage of the pandemic. For instance in early stage of the pandemic with higher number of susceptible people this may be more effective and later in the pandemic when susceptible people are very few this measure may not be cost effective.***

We appreciate the reviewer’s comments. We have added a paragraph in the discussion listing factors that can contribute to the cost-effectiveness and effectiveness of rapid testing and response.

***Reviewer #2:***

***The study is a well planned out one and the RE-AIM model is implemented to an acceptable level. The data is explicitly presented in toto and the analysis leads to the expected objectives. The manuscript is written a simple understandable manner and gives a reference to the limitations of the study.***

We appreciate the reviewer’s favorable comments!

***Reviewer #3:***

***After the iteration processes, you have scaled up testing and advised study participants to do self-testing.
1.There is no mention of any appraisal training through online mode to them on self testing.***

As explained in a new paragraph under Modifications to On-site Workflow in the Methods, we provided no other training but the one offer by the Testers on-site.

***2.There is no mention of number of tests done by participants (self swab samples) & their results.
Authors should give seperate details of the number of these self swab tests and their reslts as they have bearing on the results of the study..***

A new paragraph has been added to the Implementation section in the Results. We have also added language to the discussion to highlight that we have no indication that self-swabbing altered the results.

***3.What was the time elapsed in the handover of self swab assays to the recorder?***

We have included language in the methods to clarify that the swabs were immediately handed to the Testers after sampling.

***4.Can you give us the rationale behind twice a week testing.?***

We have added language in the Study Setting and Design section to clarify this point.
